# Supplementary material for: The right amygdala and migraine: Analyzing volume reduction and its relationship with symptom severity
Source: PLoS One. 2024 Apr 1;19(4):e0301543. doi: 10.1371/journal.pone.0301543 (PMC10984416; doi:10.1371/journal.pone.0301543)
Supplement: S1 File — (PDF) [file pone.0301543.s001.pdf]

S1 Table

Brain regions compared between MG patients and healthy subjects.

	Manual corrections		Freesurfer_original	
Regions	MG patients	Healthy Subjects	MG patients	Healthy Subjects
Left-Thalamus-Proper	7645 ± 708	7664 ± 797	7464±719	7664±812
Left-Caudate	3248 ± 335	3464 ± 487	3248±340	3464±495
Left-Putamen	4511 ± 475	4752 ± 533	4510±482	4751±542
Left-Hippocampus	4009 ± 283	4068 ± 332	4008±287	4068±337
Left-Accumbens-area	345 ± 84	371 ± 89	344±85	371±90
Right-Thalamus-Proper	7146 ± 621	7303 ± 692	7146±630	7303±705
Right-Caudate	3248 ± 335	3419 ± 465	3248±340	3419±473
Right-Putamen	4635 ± 462	4913 ± 602	4634±469	4913±612
Right-Hippocampus	4080 ± 805	4303 ± 360	4204±341	4303±367
Right-Accumbens-area	472 ± 67	492 ± 94	472±67	491±96
CC_Posterior	1009 ± 153	1023 ± 205	1009±155	1022±208
CC_Mid_Posterior	534 ± 88	570 ± 112	533±89	569±113
CC_Central	628 ± 157	640 ± 122	628±159	640±124
CC_Mid_Anterior	651 ± 150	657 ± 151	650±152	656±153
CC_Anterior	868 ± 129	867 ± 137	868±130	866±140

S2 Table

Brain regions compared between MG patients and healthy subjects.

	Manual corrections		Freesurfer_original	
Regions	MG patients	Healthy Subjects	MG patients	Healthy Subjects
lh_caudalanteriorcingulate_volume	2941 ± 510	2920 ± 576	2929 ±514	2921 ±586
lh_caudalmiddlefrontal_volume	6422 ± 1203	6542 ± 924	6420 ±1219	6545 ±938
lh_cuneus_volume	4024 ± 515	3925 ± 407	4025 ±523	3925 ±415
lh_entorhinal_volume	1794 ± 371	1859 ± 292	1793 ±377	1858 ±297
lh_fusiform_volume	8175 ± 1181	8573 ± 1253	8172 ±1195	8572 ±1276
lh_inferiorparietal_volume	12492 ± 1875	12986 ± 1610	12493 ±1901	12988 ±1639
lh_inferiortemporal_volume	11653 ± 1238	12293 ± 1771	11652 ±1260	12285 ±1818
lh_isthmuscingulate_volume	2576 ± 315	2486 ± 339	2575 ±319	2486 ±344
lh_lateraloccipital_volume	11727 ± 1605	12197 ± 1012	11731 ±1635	12193 ±1029
lh_lateralorbitofrontal_volume	8385 ± 873	8401 ± 805	8387 ±885	8399 ±821
lh_lingual_volume	5988 ± 825	6271 ± 905	5990 ±842	6272 ±921
lh_medialorbitofrontal_volume	4392 ± 508	4357 ± 522	4392 ±515	4357 ±530
lh_middletemporal_volume	13764 ± 1721	14138 ± 1911	13762 ±1747	14139 ±1947
lh_parahippocampal_volume	2078 ± 288	2101 ± 223	2077 ±291	2100 ±226
lh_paracentral_volume	4204 ± 550	4406 ± 543	4203 ±560	4405 ±553
lh_parsopercularis_volume	3943 ± 533	4053 ± 677	3943 ±544	4052 ±689
lh_parsorbitalis_volume	2007 ± 278	2025 ± 199	2008 ±283	2023 ±204
lh_parstriangularis_volume	4259 ± 623	4027 ± 695	4259 ±631	4027 ±708
lh_pericalcarine_volume	1776 ± 306	1877 ± 262	1776 ±311	1877 ±267
lh_postcentral_volume	10433 ± 1304	10996 ± 1365	10432 ±1324	10995 ±1391
lh_posteriorcingulate_volume	3430 ± 498	3366 ± 564	3432 ±509	3365 ±575
lh_precentral_volume	12835 ± 2729	13624 ± 1509	13140 ±1582	13624 ±1536
lh_precuneus_volume	9519 ± 1185	9877 ± 1109	9519 ±1205	9878 ±1129
lh_rostralanteriorcingulate_volume	3245 ± 549	3410 ± 539	3244 ±554	3408 ±549
lh_rostralmiddlefrontal_volume	10544 ± 1648	11195 ± 1349	10546 ±1678	11193 ±1375
lh_superiorfrontal_volume	22851 ± 2899	23522 ± 2997	22860 ±2940	23522 ±3053
lh_superiorparietal_volume	10892 ± 1367	11121 ± 1352	10888 ±1385	11120 ±1378
lh_superiortemporal_volume	16092 ± 1775	16919 ± 1769	16093 ±1801	16918 ±1803
lh_supramarginal_volume	9890 ± 1275	10279 ± 2581	9887 ±1293	10582 ±1710
lh_transversetemporal_volume	1118 ± 198	1173 ± 196	1117 ±201	1172 ±199
lh_insula_volume	5916 ± 592	6146 ± 617	5915 ±601	6146 ±628

S3 Table

Brain regions compared between MG patients and healthy subjects.

	Manual correction		Freesurfer_original	
Regions	MG patients	Healthy Subjects	MG patients	Healthy Subjects
rh_caudalanteriorcingulate_volume	2166 ± 470	2314 ± 506	2166 ±478	2313 ±515
rh_caudalmiddlefrontal_volume	5880 ± 890	6045 ± 1029	5880 ±905	6044 ±1049
rh_cuneus_volume	4056 ± 681	4067 ± 515	4054 ±691	4067 ±525
rh_entorhinal_volume	1747 ± 272	1891 ± 245	1746 ±276	1887 ±251
rh_fusiform_volume	8044 ± 859	8092 ± 1049	8044 ±872	8093 ±1066
rh_inferiorparietal_volume	13728 ± 1889	14694 ± 2195	13732 ±1913	14691 ±2237
rh_inferiortemporal_volume	11836 ± 1551	12507 ± 1536	11835 ±1575	12502 ±1575
rh_isthmuscingulate_volume	2418 ± 371	2451 ± 425	2417 ±377	2451 ±433
rh_lateraloccipital_volume	11768 ± 1299	12100 ± 1416	11767 ±1311	12102 ±1441
rh_lateralorbitofrontal_volume	8382 ± 784	8522 ± 896	8387 ±801	8521 ±913
rh_lingual_volume	6326 ± 829	6717 ± 891	6326 ±843	6715 ±905
rh_medialorbitofrontal_volume	4125 ± 445	4182 ± 518	4123 ±450	4184 ±526
rh_middletemporal_volume	13545 ± 1555	14120 ± 1710	13542 ±1579	14119 ±1743
rh_parahippocampal_volume	1932 ± 279	2046 ± 203	1933 ±279	2047 ±204
rh_paracentral_volume	4114 ± 491	4256 ± 462	4113 ±491	4257 ±470
rh_parsopercularis_volume	4326 ± 782	4481 ± 742	4321 ±790	4481 ±756
rh_parsorbitalis_volume	2110 ± 279	2233 ± 317	2108 ±284	2233 ±322
rh_parstriangularis_volume	4090 ± 736	4092 ± 856	4091 ±741	4092 ±871
rh_pericalcarine_volume	2106 ± 387	2153 ± 322	2106 ±392	2152 ±327
rh_postcentral_volume	9830 ± 1235	10499 ± 1296	9827 ±1253	10499 ±1320
rh_posteriorcingulate_volume	3269 ± 826	3351 ± 520	3364 ±596	3351 ±529
rh_precentral_volume	12049 ± 1558	12486 ± 1451	12049 ±1583	12484 ±1480
rh_precuneus_volume	9984 ± 2118	10392 ± 1317	10300 ±1146	10392 ±1342
rh_rostralanteriorcingulate_volume	2378 ± 439	2273 ± 493	2374 ±441	2271 ±504
rh_rostralmiddlefrontal_volume	10748 ± 2737	11371 ± 2095	11055 ±1978	11370 ±2135
rh_superiorfrontal_volume	25229 ± 3103	26241 ± 3373	25226 ±3158	26240 ±3437
rh_superiorparietal_volume	10630 ± 2527	10911 ± 1399	10938 ±1692	10909 ±1425
rh_superiortemporal_volume	15426 ± 1390	15751 ± 1491	15426 ±1415	15758 ±1512
rh_supramarginal_volume	9547 ± 1331	9442 ± 1181	9545 ±1347	9444 ±1201
rh_transversetemporal_volume	891 ± 182	914 ± 152	889 ±186	913 ±155
rh_insula_volume	6101 ± 616	6251 ± 685	6099 ±626	6251 ±697

S4 Table

Brain regions compared between MG patients and healthy subjects.

	Manual corrections		Freesurfer_original	
Regions	MG patients	Healthy Subjects	MG patients	Healthy Subjects
lh_G_cuneus_volume	2727 ± 309	2701 ± 254	2729 ±314	2703 ±257
lh_G_front_inf-Opercular_volume	3068 ± 389	3177 ± 502	3071 ±394	3181 ±508
lh_G_front_inf-Orbital_volume	1085 ± 147	1147 ± 186	1085 ±149	1144 ±188
lh_G_front_inf-Triangul_volume	2536 ± 451	2360 ± 493	2535 ±454	2356 ±499
lh_G_front_middle_volume	9508 ± 1428	9976 ± 1343	9517 ±1453	9984 ±1365
lh_G_front_sup_volume	16566 ± 2236	16977 ± 2436	16571 ±2281	16977 ±2482
lh_G_Ins_lg&S_cent_ins_volume	1483 ± 162	1508 ± 248	1487 ±160	1508 ±253
lh_G_insular_short_volume	2151 ± 232	2302 ± 266	2145 ±235	2300 ±271
lh_G_occipital_middle_volume	4890 ± 904	5033 ± 625	4892 ±918	5029 ±632
lh_G_occipital_sup_volume	2476 ± 390	2478 ± 342	2474 ±395	2475 ±354
lh_G_oc-temp_lat-fusifor_volume	4460 ± 683	4584 ± 878	4460 ±691	4582 ±892
lh_G_oc-temp_med-Lingual_volume	4114 ± 995	4452 ± 641	4236 ±677	4453 ±652
lh_G_oc-temp_med-Parahip_volume	3419 ± 596	3489 ± 544	3419 ±605	3491 ±552
lh_G_orbital_volume	5710 ± 691	5707 ± 589	5715 ±697	5708 ±598
lh_G_pariet_inf-Angular_volume	5904 ± 933	6144 ± 870	5908 ±955	6140 ±892
lh_G_pariet_inf-Supramar_volume	6376 ± 910	6857 ± 1250	6389 ±953	6861 ±1266
lh_G_parietal_sup_volume	6093 ± 989	6174 ± 848	6089 ±997	6171 ±865
lh_G_postcentral_volume	3848 ± 671	4092 ± 729	3849 ±684	4093 ±741
lh_G_precentral_volume	5950 ± 751	6106 ± 785	5943 ±754	6103 ±803
lh_G_precuneus_volume	5733 ± 735	5994 ± 759	5733 ±763	5985 ±780
lh_G_rectus_volume	2150 ± 234	2259 ± 279	2150 ±240	2255 ±284
lh_G_subcallosal_volume	1139 ± 361	1142 ± 412	1141 ±370	1137 ±418
lh_G_temp_sup-G_T_transv_volume	1008 ± 217	1079 ± 233	1006 ±223	1078 ±238
lh_G_temp_sup-Lateral_volume	5560 ± 745	5920 ± 772	5562 ±754	5918 ±788
lh_G_temp_sup-Plan_polar_volume	1908 ± 281	1883 ± 295	1909 ±284	1882 ±300
lh_G_temp_sup-Plan_tempo_volume	1820 ± 420	1964 ± 389	1818 ±427	1966 ±393
lh_G_temporal_inf_volume	6904 ± 917	7320 ± 1301	6908 ±930	7317 ±1329
lh_G_temporal_middle_volume	7539 ± 1105	7697 ± 1207	7533 ±1110	7683 ±1231
lh_Pole_occipital_volume	2821 ± 430	3010 ± 406	2824 ±439	3012 ±411
lh_Pole_temporal_volume	5536 ± 608	5623 ± 776	5532 ±617	5619 ±793

S5 Table

Brain regions compared between MG patients and healthy subjects.

	Manual corrections		Freesurfer_original	
Regions	MG patients	Healthy Subjects	MG patients	Healthy Subjects
rh_G_cuneus_volume	3112 ± 493	3123 ± 402	3111 ±500	3125 ±413
rh_G_front_inf-Opercular_volume	3145 ± 502	3279 ± 517	3144 ±511	3278 ±526
rh_G_front_inf-Orbital_volume	1012 ± 213	1048 ± 203	1016 ±220	1048 ±206
rh_G_front_inf-Triangul_volume	2144 ± 493	2158 ± 575	2140 ±504	2159 ±584
rh_G_front_middle_volume	9095 ± 1532	9157 ± 1646	9131 ±1591	9157 ±1677
rh_G_front_sup_volume	15410 ± 1847	15808 ± 1947	15397 ±1863	15806 ±1986
rh_G_Ins_lg&S_cent_ins_volume	1442 ± 221	1494 ± 193	1464 ±230	1494 ±196
rh_G_insular_short_volume	2062 ± 342	2160 ± 331	2039 ±342	2159 ±337
rh_G_occipital_middle_volume	4909 ± 777	5241 ± 793	4912 ±790	5245 ±805
rh_G_occipital_sup_volume	2795 ± 411	2777 ± 383	2801 ±416	2775 ±392
rh_G_oc-temp_lat-fusifor_volume	4693 ± 712	4654 ± 749	4694 ±723	4649 ±768
rh_G_oc-temp_med-Lingual_volume	4312 ± 622	4488 ± 611	4310 ±632	4484 ±617
rh_G_oc-temp_med-Parahip_volume	3402 ± 521	3546 ± 369	3404 ±524	3546 ±375
rh_G_orbital_volume	6492 ± 752	6681 ± 714	6483 ±759	6684 ±724
rh_G_pariet_inf-Angular_volume	6593 ± 1138	6968 ± 1198	6581 ±1122	6954 ±1225
rh_G_pariet_inf-Supramar_volume	5712 ± 1007	5782 ± 907	5711 ±1022	5783 ±924
rh_G_parietal_sup_volume	5212 ± 812	5027 ± 829	5205 ±824	5030 ±846
rh_G_postcentral_volume	3469 ± 535	3824 ± 667	3467 ±539	3823 ±679
rh_G_precentral_volume	5714 ± 936	5987 ± 915	5713 ±950	5984 ±935
rh_G_precuneus_volume	5362 ± 807	5365 ± 870	5367 ±829	5364 ±886
rh_G_rectus_volume	1881 ± 205	1950 ± 218	1879 ±220	1950 ±222
rh_G_subcallosal_volume	689 ± 181	736 ± 217	691 ±186	737 ±220
rh_G_temp_sup-G_T_transv_volume	851 ± 218	890 ± 161	850 ±221	890 ±163
rh_G_temp_sup-Lateral_volume	5017 ± 629	5056 ± 635	5012 ±639	5057 ±646
rh_G_temp_sup-Plan_polar_volume	2138 ± 386	2162 ± 471	2131 ±396	2164 ±476
rh_G_temp_sup-Plan_tempo_volume	1648 ± 266	1657 ± 250	1645 ±270	1658 ±252
rh_G_temporal_inf_volume	6613 ± 1132	7142 ± 1208	6604 ±1152	7135 ±1242
rh_G_temporal_middle_volume	7848 ± 1684	8464 ± 1132	8162 ±999	8463 ±1153
rh_Pole_occipital_volume	4623 ± 447	4878 ± 515	4621 ±456	4876 ±524
rh_Pole_temporal_volume	6063 ± 588	6204 ± 890	6063 ±601	6199 ±909
