# Supplementary material for: The right amygdala and migraine: Analyzing volume reduction and its relationship with symptom severity
Source: PLoS One. 2024 Apr 1;19(4):e0301543. doi: 10.1371/journal.pone.0301543 (PMC10984416; doi:10.1371/journal.pone.0301543)
Supplement: S2 File — (PDF) [file pone.0301543.s002.pdf]

S6 Table Partial correlation analysis between variables in MG patients.

Correlations

Control Variables			MIDAS	MSQ	CES-D	STAI(state)	STAI(trait)	Left-Pallidum	Left-Amygdala	Right-Pallidum	Right-Amygdala
Sex & ICV as covariates	MIDAS	Correlation	1.000								
		Significance (2-tailed)									
		df	0								
	MSQ	Correlation	.607	1.000							
		Significance (2-tailed)	.001								
		df	24	0							
	CES-D	Correlation	-.242	.365	1.000						
		Significance (2-tailed)	.234	.050							
		df	24	24	0						
	STAI(state)	Correlation	-.249	.265	.825	1.000					
		Significance (2-tailed)	.221	.191	.000						
		df	24	24	24	0					
	STAI(trait)	Correlation	-.269	.146	.743	.906	1.000				
		Significance (2-tailed)	.184	.477	.000	.000					
		df	24	24	24	24	0				
	F_Left-Pallidum	Correlation	.242	-.009	-.112	-.184	-.191	1.000			
		Significance (2-tailed)	.234	.964	.585	.367	.349				
		df	24	24	24	24	24	0			
	F_Left-Amygdala	Correlation	-.063	-.023	-.023	.002	-.089	.049	1.000		
		Significance (2-tailed)	.760	.913	.912	.992	.664	.813			
		df	24	24	24	24	24	24	0		
	F_Right-Pallidum	Correlation	.029	-.109	-.218	-.264	-.209	.676	-.228	1.000	
		Significance (2-tailed)	.889	.596	.285	.193	.305	.000	.263		
		df	24	24	24	24	24	24	24	0	
	F_Right-Amygdala	Correlation	-.403	-.248	.157	-.045	-.164	.106	.513	-.133	1.000
		Significance (2-tailed)	.041	.221	.445	.826	.423	.607	.007	.518	
		df	24	24	24	24	24	24	24	24	0

S7 Table Partial correlation analysis between variables in healthy subjects.

Correlations

Control Variables			MIDAS	MSQ	CES-D	STAI(state)	STAI(trait)	Left-Pallidum	Left-Amygdala	Right-Pallidum	Right-Amygdala
Sex & ICV as covariates	MIDAS	Correlation	1.000								
		Significance (2-tailed)									
		df	0								
	MSQ	Correlation	-.018	1.000							
		Significance (2-tailed)	.934								
		df	22	0							
	CES-D	Correlation	-.085	.320	1.000						
		Significance (2-tailed)	.692	.128							
		df	22	22	0						
	STAI(state)	Correlation	-.147	.282	.530	1.000					
		Significance (2-tailed)	.494	.182	.008						
		df	22	22	22	0					
	STAI(trait)	Correlation	-.263	.211	.640	.757	1.000				
		Significance (2-tailed)	.215	.321	.001	.000					
		df	22	22	22	22	0				
	F_Left-Pallidum	Correlation	.061	.505	.260	.465	.223	1.000			
		Significance (2-tailed)	.778	.012	.221	.022	.294				
		df	22	22	22	22	22	0			
	F_Left-Amygdala	Correlation	.285	.070	.052	.006	-.114	.258	1.000		
		Significance (2-tailed)	.178	.746	.811	.977	.596	.224			
		df	22	22	22	22	22	22	0		
	F_Right-Pallidum	Correlation	.129	.345	-.096	.338	.039	.763	.413	1.000	
		Significance (2-tailed)	.547	.099	.655	.106	.856	.000	.045		
		df	22	22	22	22	22	22	22	0	
	F_Right-Amygdala	Correlation	.230	.136	.095	.062	.005	.153	.721	.180	1.000
		Significance (2-tailed)	.280	.526	.660	.773	.983	.474	.000	.400	
		df	22	22	22	22	22	22	22	22	0
