# Supplementary material for: The right amygdala and migraine: Analyzing volume reduction and its relationship with symptom severity
Source: PLoS One. 2024 Apr 1;19(4):e0301543. doi: 10.1371/journal.pone.0301543 (PMC10984416; doi:10.1371/journal.pone.0301543)
Supplement: S3 File — (PDF) [file pone.0301543.s003.pdf]

S8 Table Direct, total and indirect effects of path analysis.

Direct effects

			Estimate	P
CESD	→	MSQ	0.58	< 0.0001
MSQ	→	MIDAS	0.71	< 0.0001
MIADS	→	R. Amygdala	-0.38	0.002

Covariates

			Estimate	P
R. Pallidum	→	CESD	-0.35	0.01
State anxiety	→	CESD	0.81	P<0.0001
R. Pallidum	→	State anixety	-0.16	0.08

Total effects

	CESD	MSQ	MIDAS
MSQ	0.58		
MIDAS	0.41	0.65	
R. Amydala	-0.16	-0.27	-0.38

Indirect effects

	CESD	MSQ	MIDAS
MSQ	0	0	0
MIDAS	0.41	0	0
R. Amydala	-0.59	-0.27	0
